# In vitro osteoblastic differentiation of mesenchymal stem cells generates cell layers with distinct properties

**DOI:** 10.1186/s13287-018-0942-x

**Published:** 2018-07-27

**Authors:** Hanna Hanna, Lluis M. Mir, Franck M. Andre

**Affiliations:** Vectorology and Anticancer Therapies, UMR 8203, CNRS, Univ. Paris-Sud, Gustave Roussy, Université Paris-Saclay, PR2, 114 rue Edouard Vaillant, 94 805 Villejuif, France

**Keywords:** Mesenchymal stem cells, Multilayers, Collagenase I, Trypsin, Extracellular matrix, Cluster of differentiations, Calcium oscillations

## Abstract

**Background:**

Differentiation of mesenchymal stem cells to osteoblasts is widely performed in research laboratories. Classical tests to prove this differentiation employ procedures such as cell fixation, cell lysis or cell scraping. Very few studies report gentle dissociation of mesenchymal stem cells undergoing an osteodifferentiation process. Here we used this technique to reveal the presence of several cell layers during osteogenesis and to study their different properties.

**Methods:**

Through the sequential enzymatic detachment of the cells, we confirm the presence of several layers of differentiated cells and we compare them in terms of enzymatic sensitivity for dissociation, expression of cluster of differentiation, cytosolic calcium oscillations and osteogenic potential. Adipogenic and neurogenic differentiations were also performed in order to compare the cell layers.

**Results:**

The cells undergoing differentiation formed one layer in the neurogenic differentiation, two layers in the adipogenic differentiation and at least four layers in the osteogenic differentiation. In the latter, the upper layers, maintained by a collagen I extracellular matrix, can be dissociated using collagenase I, while the remaining lowest layer, attached to the bottom of the dish, is sensitive only to trypsin-versene. The action of collagenase I is more efficient before the mineralization of the extracellular matrix. The collagenase-sensitive and trypsin-sensitive layers differ in their cluster of differentiation expression. The dissociation of the cells on day 15 reveals that cells could resume their growth (increase in cell number) and rapidly differentiate again in osteoblasts, in 2 weeks (instead of 4 weeks). Cells from the upper layers displayed a higher mineralization.

**Conclusions:**

MSCs undergoing osteogenic differentiation form several layers with distinct osteogenic properties. This could allow the investigators to use upper layers to rapidly produce differentiated osteoblasts and the lowest layer to continue growth and differentiation until an ulterior dissociation.

**Electronic supplementary material:**

The online version of this article (10.1186/s13287-018-0942-x) contains supplementary material, which is available to authorized users.

## Background

Mesenchymal stem cell (MSC) osteogenic differentiation was the first differentiation to be identified by Friedenstein et al. in the late 1960s–early 1970s [1, 2]. As the MSCs (known then as marrow stromal cells) were extracted from the bone marrow, it was not surprising that they could give rise to osteoblasts. In vitro, osteogenic differentiation occurs during a period of 1 month and produces differentiated osteoblasts. Downregulation of DNA replication by the second week of differentiation is associated with expression of osteoblast markers (such as alkaline phosphatase (ALP)), processing of type I procollagen to collagen I under the effect of ascorbic acid, and progressive deposition of a collagenous extracellular matrix (ECM) [3]. Later on, other bone markers can be detected, such as osteocalcin, osteopontin, bone sialoprotein and osteonectin. Hydroxyapatite deposits mark the final phase of osteoblast phenotypic development [3]. The ECM is an essential element for the osteogenic differentiation; it is secreted by the MSCs undergoing osteogenic differentiation and contains growth factors and many proteins such as fibronectin, vitronectin, laminin, osteopontin and osteonectin [4]. Osteoblasts must be in contact with a collagen I matrix before they can differentiate [5]. ECM formation is a marker of MSC differentiation to osteoblast, and it accumulates maximally after 1 week of culture. After 2 weeks of culture, mineralization of ECM begins, which marks the final phase of the osteoblast phenotypic development [3]. Cell proliferation before mineralization is a critical process for increasing bone mass. Because the cells are exposed to the differentiating agents when they form a more or less confluent monolayer in the culture dishes, the differentiated cells in 2D cultures are sometimes referred to as “monolayers” [6], “confluent monolayers” [7] or “cells in monolayers” [8]. The term “multilayer” was barely used [9]. Zhu et al. [10] showed that weak osteogenesis is characterized by the formation of an ALP-positive cell monolayer while strong osteogenesis is characterized by the presence of multilayered ALP-positive nodular structures. To assess the differentiation potential, the investigators used many classical tests that label the cells or measure the osteogenic markers. In all of these methods, there was no dissociation of the cells from the different layers. Indeed, the first step is often the fixation of the cells, for example to stain the cells with Alizarin Red that labels the deposits of calcium phosphate (hydroxyapatite Ca_5_(PO_4_)_3_(OH)) [11, 12]. Other tests such as immunofluorescent labeling [13, 14], ECM protein production [4] and external ALP measurement [12, 15] are performed without dissociation of the cells, just after their fixation. In other methods, the supernatant of the cells is taken to measure osteocalcin [6, 16], type I procollagen [16], bone sialoprotein or different specific cytokine production [17, 18] by ELISA or other techniques. Finally, other methods start with the scraping or the lysis of the cells using trichloroacetic acid and SDS [9], sonication [16], trizol [19], RIPA buffer [17] or many freeze–thaw cycles [15] in order to perform DNA and calcium assays [16, 20, 21], to measure internal ALP by colorimetric assays [14, 15, 17, 21, 22] or to detect specific proteins by western blot analysis or mRNA by RT-PCR [6, 8, 9, 12]. None of these techniques dissociated the living cells. Some studies [14, 23] used trypsin alone (until day 14 of osteodifferentiation, before the mineralization occurred) or trypsin–EDTA [24, 25] to harvest all of the layers in 2D cultures.

In our study, we used human adipose mesenchymal stem cells (haMSCs) to perform osteogenic, adipogenic and neurogenic differentiations to assess the multipotency of the haMSCs and to compare the evolution of the cells in one or several layers during the differentiation. Confocal microscopy observations were performed in order to assess the evolution of the cells in one or many layers in the three differentiations. Furthermore, differential dissociation by collagenase I and trypsin was performed on the layers in osteogenic differentiation. The different layers were then characterized by their cluster of differentiation markers and their Ca^2+^ oscillations in order to evaluate whether they were identical or not. The different cell populations resulting from differential dissociation at day 15 were also cultivated separately to evaluate their osteogenic potential. This study presents new observations on the cell layers in an in-vitro osteogenic differentiation and proposes a new method to dissociate the cells, which could expand their use and facilitate applications.

## Methods

### Cell culture and differentiation

haMSCs were isolated from surgical waste of individuals undergoing elective lipoaspiration. The cells were grown in Dulbecco’s Modified Eagle Medium (DMEM) supplemented with 10% foetal bovine serum, 100 U/ml penicillin and 100 mg/ml streptomycin, and were cultured at 37 °C in a humidified incubator with 5% CO_2_. Cells were passed twice a week (every passage corresponded to one doubling time of the population). The multipotency capabilities of the cells were assessed by submitting them to differentiation conditions as previously reported by Liew et al. [26]*.* The cell culture chemicals were purchased from Fischer Scientific (Parc d’innovation, Illkirch, France). Prior to every differentiation, cells were seeded at a density of 15,000 cells/cm^2^ and left in culture for 2–3 days to attain confluence, after which the normal medium was removed and differentiation medium was added. This medium change corresponded to differentiation day 1. The osteogenic medium was composed of complete alpha MEM supplemented with 100 nM of dexamethasone, 200 μM of ascorbic acid and 10 mM of glycerol 2-phosphate. The medium was changed twice a week. For the adipogenic differentiation, two media were consecutively used: an induction medium composed of complete DMEM supplemented with 1 μM dexamethasone, 200 μM indomethacin, 500 μM 3-isobutyl-1-methylxantine and 10 μg/ml insulin for 2–3 days; and a maintenance medium composed of complete DMEM supplemented with 10 μg/ml insulin renewed every 24 h. For the neurogenic differentiation, a ready-to-use neurogenic induction medium was used from Promocell (C-28015), and was changed every 48 h. The controls were haMSCs cultivated without passage in their normal medium, which was changed twice a week.

### Cell dissociation and counting

In adipogenic differentiation and neurogenic differentiation, cells were simply trypsinized and counted three times at every time point (days 1, 8, 15, 22 and 29). As described in this article, several layers of cells could be distinguished in osteogenic differentiation. To dissociate the upper layers before the calcium deposits begun to appear, 2 mg/ml collagenase I (Fisher Scientific, Illkirch, France) diluted in PBS was added to the cells for 30 min. After collagenase I action, the cell cultures were pipetted gently to remove all cells of the upper layers. The remaining layer was trypsinized. When the mineralization occurred (i.e. when Ca^2+^ deposits became apparent), the calcium deposits were removed using 20–40 mM of EDTA in PBS for 20–40 min, depending on the density of these deposits. The cells of the upper layers were then detached using a higher concentration of collagenase (4 mg/ml) and the cells of the lowest layer using trypsin. The cells from all the layers were gathered together in one tube and counted three times at every time point.

### Alkaline phosphatase assay and Alizarin Red staining

In order to test the ALP activity (osteogenic differentiation), cells were harvested using collagenase I and trypsin, centrifuged at 1500 rpm for 5 min, resuspended in 1 ml of cold PBS (4 °C) and counted. A volume corresponding to 300,000 cells was taken, centrifuged and suspended in 600 μl of assay buffer (Abcam), and centrifuged at 13,200 rpm for 5 min at 4 °C. The supernatant was transferred to a new tube. Then 200 μl of the supernatant was distributed in four wells of a 96-well plate (50 μl/well). One of these wells served to determine the background through the simultaneous addition of 80 μl *p*-nitrophenyl phosphate (pNPP) 1 mM and 20 μl of stop solution. For the three remaining wells, 80 μl of pNPP was added. After 60 min, 20 μl of stop solution (Abcam) was added to the wells and the plate was read in a spectrophotometer (BioTek, Colmar, France) to measure the optical density at 405 nm. The quantity of *p*-nitrophenol (pNP) in each well was determined using a standard curve established using pNPP and purified ALP enzyme. Then 350 μl of the remaining supernatant was incubated with 750 nM of Hoechst 33342 for 30 min and distributed in triplicate in an opaque 96-well plate to read the fluorescence at 365 nm excitation. Prior to Alizarin Red staining, cells were washed in PBS and fixed in 95% methanol for 10 min. Alizarin Red S solution 2% was added for 5 min, then rinsed with water and imaged under an epifluorescence microscope to visualize the Ca^2+^ deposits. To quantify the Alizarin Red staining, 10% cetylperidinium chloride (Sigma Aldrich) was added (1 ml for 10 cm^2^) and cells were incubated for 20 min to elute the stain. Alizarin Red staining was then quantified by measuring the absorbance of the eluted stain at 570 nm using a spectrophotometer and normalizing by the number of cells.

### FABP4 and Bodipy staining

To assess adipogenic differentiation, the attached cells were washed after medium removal, fixed in 10% formalin for 20 min and washed three times in PBS + 1% BSA. The cells were then permeabilized and blocked with PBS containing 0.3% Triton X-100, 1% BSA and 10% universal blocking reagent. After blocking, the cells were incubated overnight at 4 °C with an antibody (PA5-30591; Fisher Scientific) against fatty acid binding protein 4 (FABP4). The cells were then washed three times in PBS + 1% BSA, incubated for 60 min in the dark with a secondary antibody coupled to Alexa Fluor 488 (A-11008; Fisher Scientific), washed three times in PBS + 1% BSA and visualized under an epifluorescence microscope (Zeiss Axiovert S100; Carl Zeiss, Oberkochen, Germany).

In order to stain the cells with Bodipy, the cell medium was removed and the cells were incubated for 1 h with a fresh medium containing 100 μg/ml of Bodipy (Molecular Probes, Fisher Scientific), washed and imaged under the epifluorescence microscope.

### MAP2 and cresyl violet staining

To assess neurogenic differentiation, cells were fixed and incubated with microtubule-associated protein 2 (MAP2) antibody (reference 4542; Cell signaling, Saint Quentin, France) following the same protocol as for the FABP4 antibody (see earlier).

In order to stain Nissl bodies, cells were washed and fixed with 10% formalin for 20 min. Then a 0.5% cresyl violet staining solution (Sigma Aldrich) was added to cells and incubated for 30 min at room temperature. The cells were then washed three times in PBS and imaged under an epifluorescence microscope.

### Living cell imaging

haMSCs were seeded, prior to the differentiation, on a glass-bottom, four-well μ-slide (reference 80,447; Ibidi GMBH, Planegg, Germany). Prior to confocal imaging, cells were incubated for 30 min with 375 nM Hoechst 33342 (nuclei marker; λ_exc_ = 480 nm, λ_em_ = 535 nm) and 5 μM of fluorescein diacetate (FDA) (cytoplasm marker; λ_exc_ = 405 nm, λ_em_ = 486 nm). A confocal microscope Leica TCS SPE with an objective HC PL APO CS2 20× and LAS AF software version 3.3 (Leica, Germany) were used to visualize the mono/multilayers in osteogenic, adipogenic or neurogenic differentiation.

### Calcium oscillation visualization and analysis

The upper layers in osteogenic differentiation were dissociated by collagenase I as already described, the lowest layer was trypsinized and the cells from each layer type were seeded apart at a density of 15,000 cells/cm^2^ for 24 h in an osteogenic differentiation medium. After 24 h, the medium was removed and the cells were incubated for 30 min with 5 μM of Fluo-4 AM (Fischer Scientific), a fluorescent Ca^2+^ marker, in a humidified 5% CO_2_ atmosphere at 37 °C in osteogenic medium. The incubation buffer also contained 375 nM of the nuclear fluorescent dye Hoechst 33342. After incubation, the attached cells were rinsed three times with PBS and 500 μl of fresh medium was added to the cells. Images of the cells were then taken every 10 s for 30 min under a fluorescence microscope (Zeiss Axiovert 100 with a Zeiss AxioCam Hrc camera controlled by Axio Vision 4.6 software; Carl Zeiss). The excitation and emission wavelengths used for Fluo-4 were 496 nm and 515 nm respectively. The nuclear dye Hoechst 33342 (λ_ex_ = 350 nm, λ_em_ = 461 nm) was used to facilitate cell identification and calcium oscillation determination using Cell Profiler (version 2.0) software (Broad Institute, Cambridge, MA, USA).

### Immunofluorescent staining

Upper layers in osteogenic differentiation were dissociated with 2 mg/ml of collagenase I. The remaining layer was trypsinized. On day 22, the differentiating cell cultures were treated first by 20 mM EDTA in PBS before collagenase addition. Cells were counted and centrifuged at 825 rpm for 10 min, after which the supernatant was aspirated and the cells were suspended in a buffer of PBS, 0.5% BSA and 2 mM EDTA (100 μl/100,000 cells). Then 10 μl of anti-CD44 APC, CD90 PE and CD105 FITC was added per 100 μl of cells. The cells were incubated with the antibodies for 10 min at 4 °C in the dark. The cells were then washed with 1 ml of buffer and centrifuged. The pellet was resuspended in 300 μl of buffer for analysis by flow cytometry (Accuri C6 cytometer; BD Life Sciences, Le Pont de Claix, France). The antibodies were purchased from Miltenyi Biotec (Paris, France).

### Statistical analysis

**Fig. 1:**
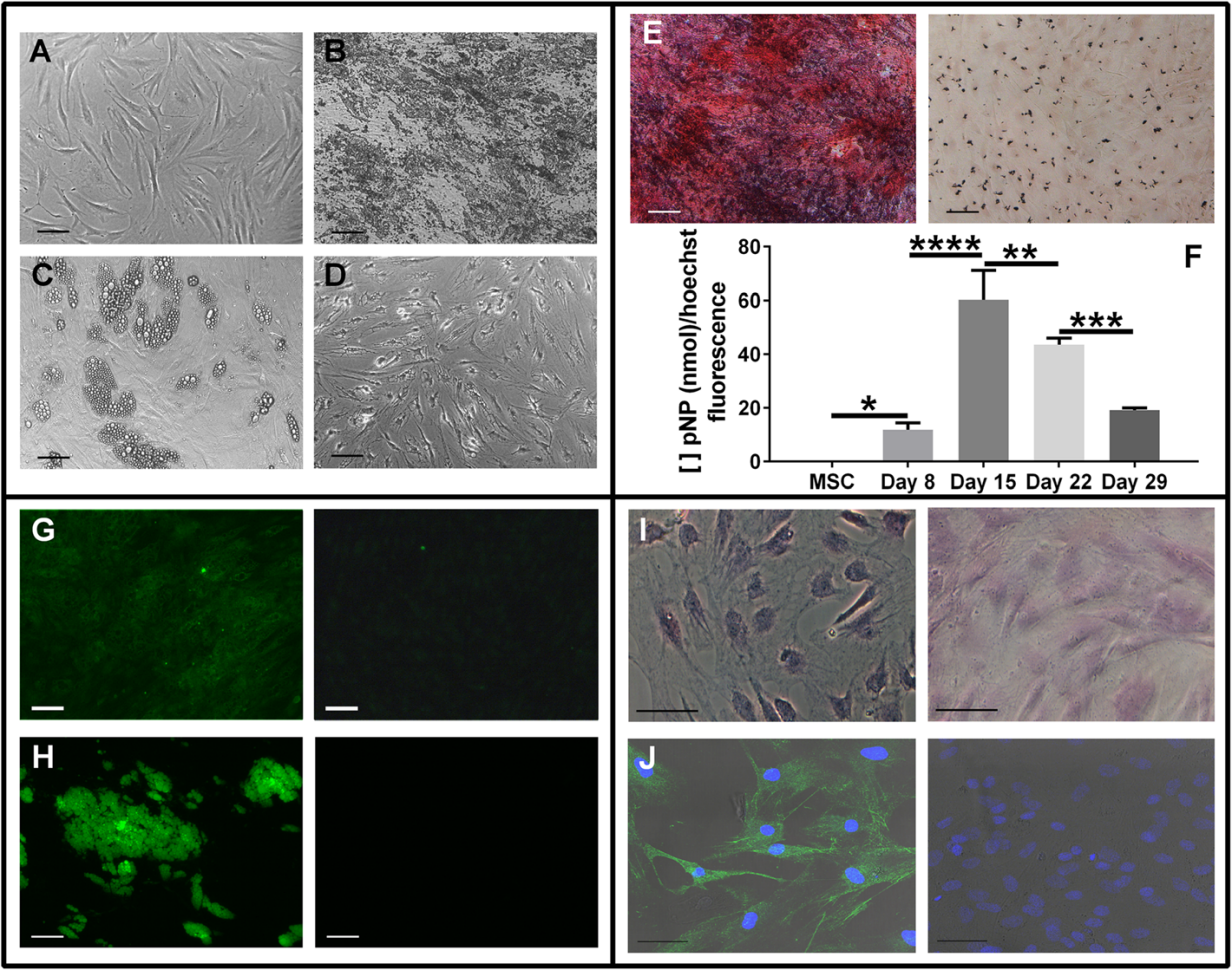
haMSC differentiation. **a–-d** Morphological aspects of the cells. (**a**) Control haMSCs (grown in classical MSC culture medium) after 3 days of culture. (**b**), (**c**), (**d**) haMSCs undergoing respectively osteogenic (day 29), adipogenic (day 15) and neurogenic (day 5) differentiation. **e**, **f** Osteogenic differentiation characterized by Alizarin Red staining (**e**) and alkaline phosphatase activity (**f**). **g**, **h** Adipogenic differentiation characterized by labeling with FABP4 antibody (**g**) and bodipy staining (**h**). **i**, **j** Neurogenic differentiation characterized by cresyl violet staining (**i**) and labeling with MAP2 antibody and Hoechst (**j**). In (**e, g–j**), right photograph displays background staining of undifferentiated control haMSCs cultured in classical growth medium. Calibration bars correspond to 100 μm. ****p < 0.0001, ***p < 0.001, **p < 0.01, *p < 0.05. pNP *p*-nitrophenol, MSC mesenchymal stem cell

To compare the differences in alkaline phosphatase activity during osteogenesis (Fig. 1), one-way ANOVA with Tukey’s multiple comparisons test was used. To compare the difference in immunofluorescent staining between the upper and lowest layers (Fig. 5a), an unpaired *t* test was used. To analyze the influence of the layers and the number of days of differentiation on Ca^2+^ oscillations (Fig. 5b), two-way ANOVA with Tukey’s multiple comparisons test was used. To compare the control to the other conditions, we used one-way ANOVA with Dunnett’s multiple comparisons test. All results are expressed as the mean and standard deviation from three different differentiations. These three differentiations were performed using cells from three different donors.

## Results

### Characterization of cell differentiation

Before analyzing the layers formed in various haMSC differentiations, we first confirmed that the cells had undergone every differentiation correctly. Characterization of osteodifferentiation was performed by the measurement of ALP activity and the staining of Ca^2+^ deposits using Alizarin Red. The undifferentiated control haMSCs (Fig. 1a) were cultured in classical MSC growth medium and displayed no Alizarin Red labeling and no ALP activity (Fig. 1e right, f). In osteogenic differentiation, the Ca^2+^ deposits started to accumulate at the end of the second week and became more and more dense (Fig. 1b). These deposits were colored red by the Alizarin Red staining (Fig. 1e left). The ALP activity slightly increased at day 8 of osteodifferentiation and reached a peak on day 15 (Fig. 1f).

The adipodifferentiation was characterized by FABP4 staining and lipidic vacuole formation. The lipidic vacuoles shown in Fig. 1c are stained green by Bodipy staining (Fig. 1h left). The adipose cells fluoresced in green after FABP4 labeling (Fig. 1g left). The undifferentiated haMSC controls (cultured in classical MSC growth medium) displayed no Bodipy staining (Fig. 1h right) or FABP4 antibody labeling (Fig. 1g right). The cresyl violet that stains the Nissl bodies specific of the neuronal differentiation (Fig. 1d) labeled the haMSCs undergoing neurodifferentiation (Fig. 1i left) but not the undifferentiated haMSC controls (Fig. 1i right). The MAP2 antibody interacted with neuronal cells but not with undifferentiated haMSC controls, and this was revealed by the green fluorescence displayed by the neuronal cells (Fig. 1j).

### Evolution of the cell number in the different differentiation pathways

**Fig. 2:**
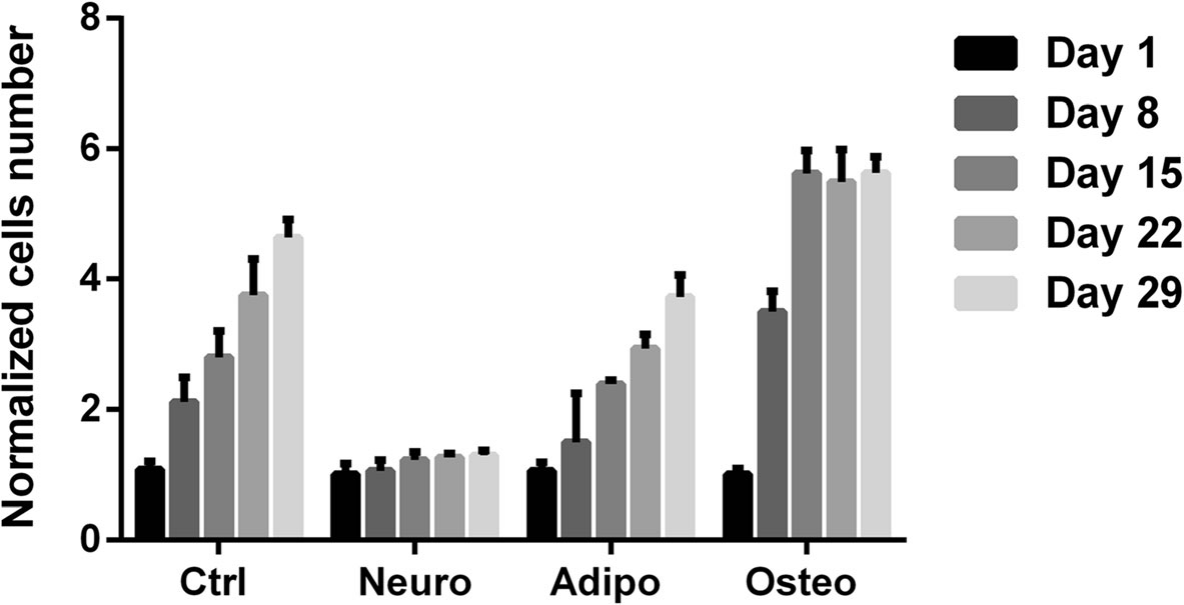
Evolution of cell number between day 1 and day 29 in different groups of cells. Controls are undifferentiated haMSCs cultured in classical growth medium. Ctrl control, Neuro neurogenic, Adipo adipogenic, Osteo osteogenic

To better understand the various numbers of cell layers observed between the different differentiation pathways, the kinetics of cell proliferation were assessed. When the cells were exposed to the neurodifferentiation medium, they showed no proliferation at all: the cell number remained nearly the same during all of the differentiation process (Fig. 2). In adipogenic differentiation, cell proliferation was maintained, only slightly reduced with respect to the proliferation in the controls (Fig. 2). On the contrary, in osteogenic differentiation there was a real stimulation of the cell proliferation: cells proliferated extensively between day 1 and 15 and faster than the control haMSCs (Fig. 2). At days 8 and 15, there were nearly four and six times more cells than at day 1. After day 15, no further increase of the cell number was observed.

**Fig. 3:**
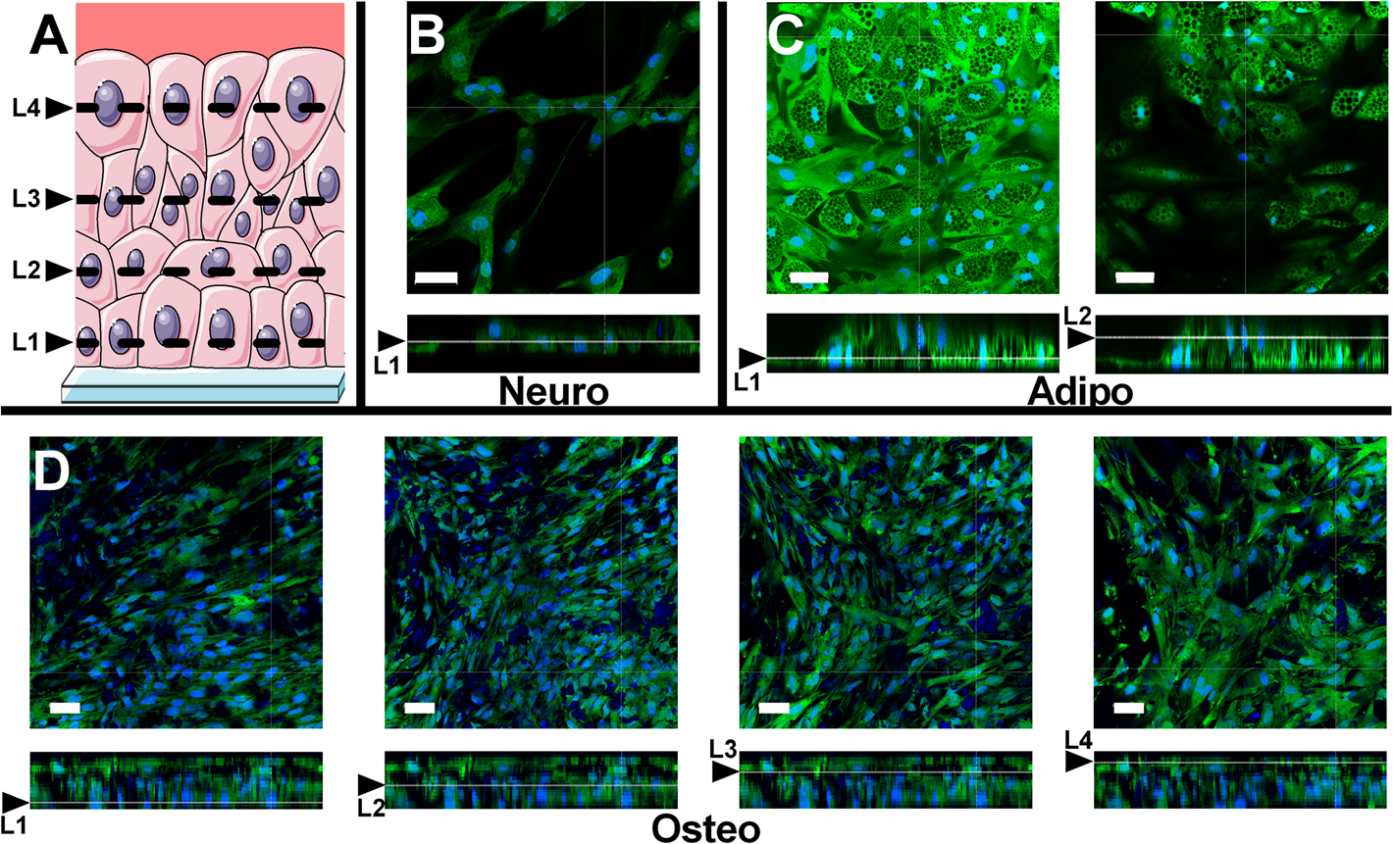
**a** Schematic representation of different layers of differentiation observed using confocal microscopy. Different layers of neurogenic (**b**), adipogenic (**c**) and osteogenic (**d**) differentiation. Confocal observation at day 22, stained with Hoechst and fluorescein diacetate. For each differentiation, panels represent (from left to right) bottom layer (L1, layer in contact with the coverslide), second (L2), third (L3) and fourth (L4) layer, respectively. Grids below represent position of confocal slice on *Z* axis. Calibration bars correspond to 50 μm. Neuro neurogenic, Adipo adipogenic, Osteo osteogenic

In osteogenic differentiation, due to the initial very rapid cell proliferation, cells started to accumulate in several layers in the first week of osteodifferentiation. The number of layers stabilized at the end of the second week when the cells stopped proliferation. Four different layers of cells could be detected using confocal microscopy on day 22 of osteodifferentiation, every layer hiding the previous one (Fig. 3d). The layers were well separated, and almost no cell could be observed in between. In adipogenic differentiation (Fig. 3c), above a first continuous layer, a discontinuous second layer of cells could also be observed in some parts of the dish. In neurogenic differentiation, even at day 22, cells always remained in one single layer (Fig. 3b) in agreement with the total absence of cell proliferation (Fig. 2). Furthermore, the cells shrank with time when they transformed from the mesenchymal fibroblastic shape to the neuronal one.

### Characterization of the different cell layers in osteogenic differentiation

**Fig. 4:**
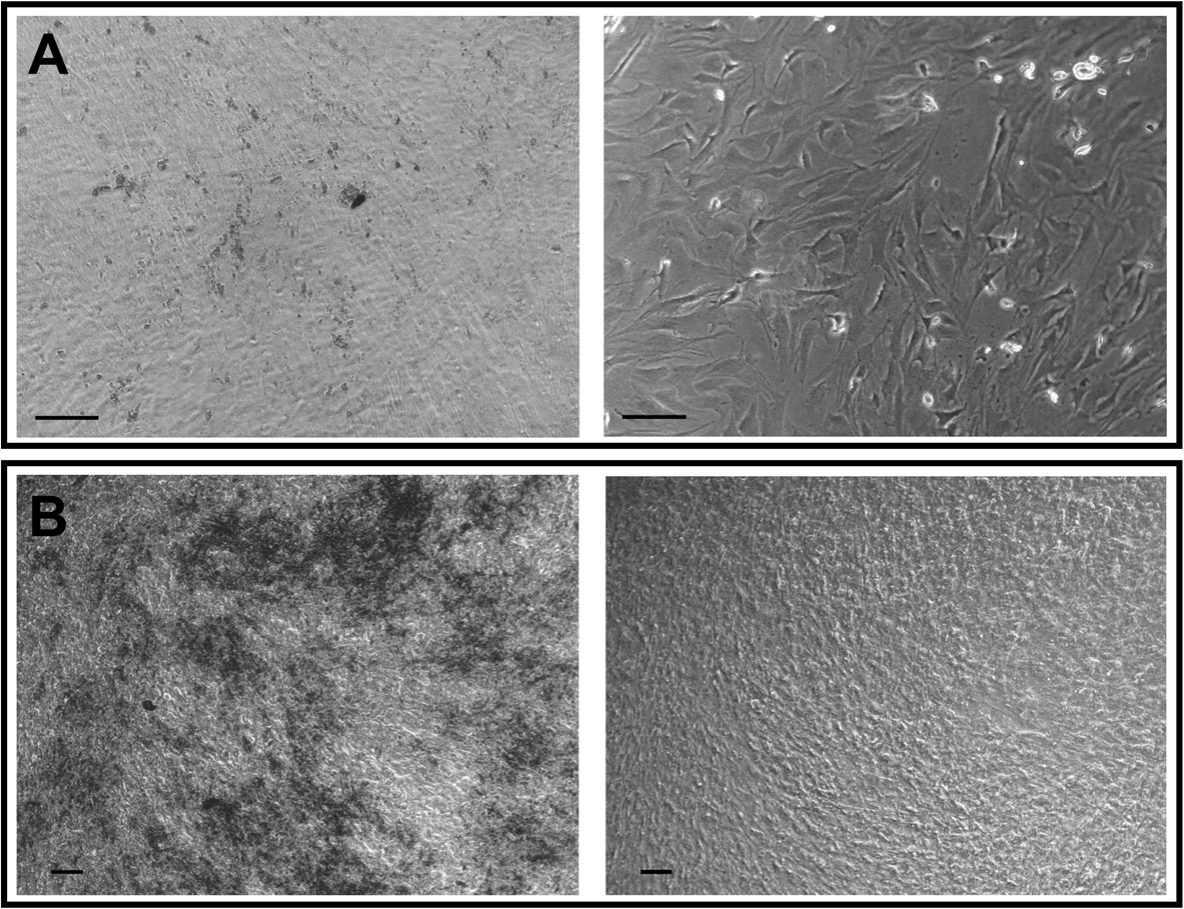
Action of collagenase I and EDTA on osteogenic layers. **a** Observation of cells before (left) and after (right) action of collagenase I (2 mg/ml) on day 15 of osteodifferentiation. **b** Ca^2+^ deposits before (left) and after (right) action of 40 mM of EDTA on day 29 of osteodifferentiation. Calibration bars correspond to 100 μm

When classical trypsinization (incubation of up to 10 min) was performed on the cells undergoing osteogenic differentiation, trypsin had absolutely no effect. It was only after more than 30 min of incubation with trypsin that all of the layers started to detach altogether as a whole surface (like a rolled-up carpet) with all of the layers still attached together, not detaching as individual cells or individual layers. This means that trypsin is only able to remove the connections between the bottom layer and the culture dish, not the connections between layers or the connections between cells within a given layer. On the contrary, when 2 mg/ml collagenase I was applied to the cells undergoing an osteogenic differentiation, the bottom cells remained attached to the culture dish and all of the cells from the upper layers were individually dissociated (not as whole intact layers). This demonstrates that collagenase I only removed interactions between individual cells but not the connections between the bottom cells and the culture dish. Moreover, after the action of collagenase I, only the bottom layer remained attached (Fig. 4a right). This lowest layer was not sensitive to collagenase. It could only be detached by trypsin. Therefore, collagenase I was able to selectively detach all of the cells from the upper layers but not those from the bottom layer, and trypsin was doing the exact opposite.

When the mineralization occurred, collagenase I had no effect at all, even on the upper layers. The Ca^2+^ deposits had to be removed first. Prior EDTA treatment (20–40 mM for 20–40 min) was used to remove these deposits and the collagenase concentration had to be increased from 2 to 4 mg/ml (Fig. 4b).

**Fig. 5:**
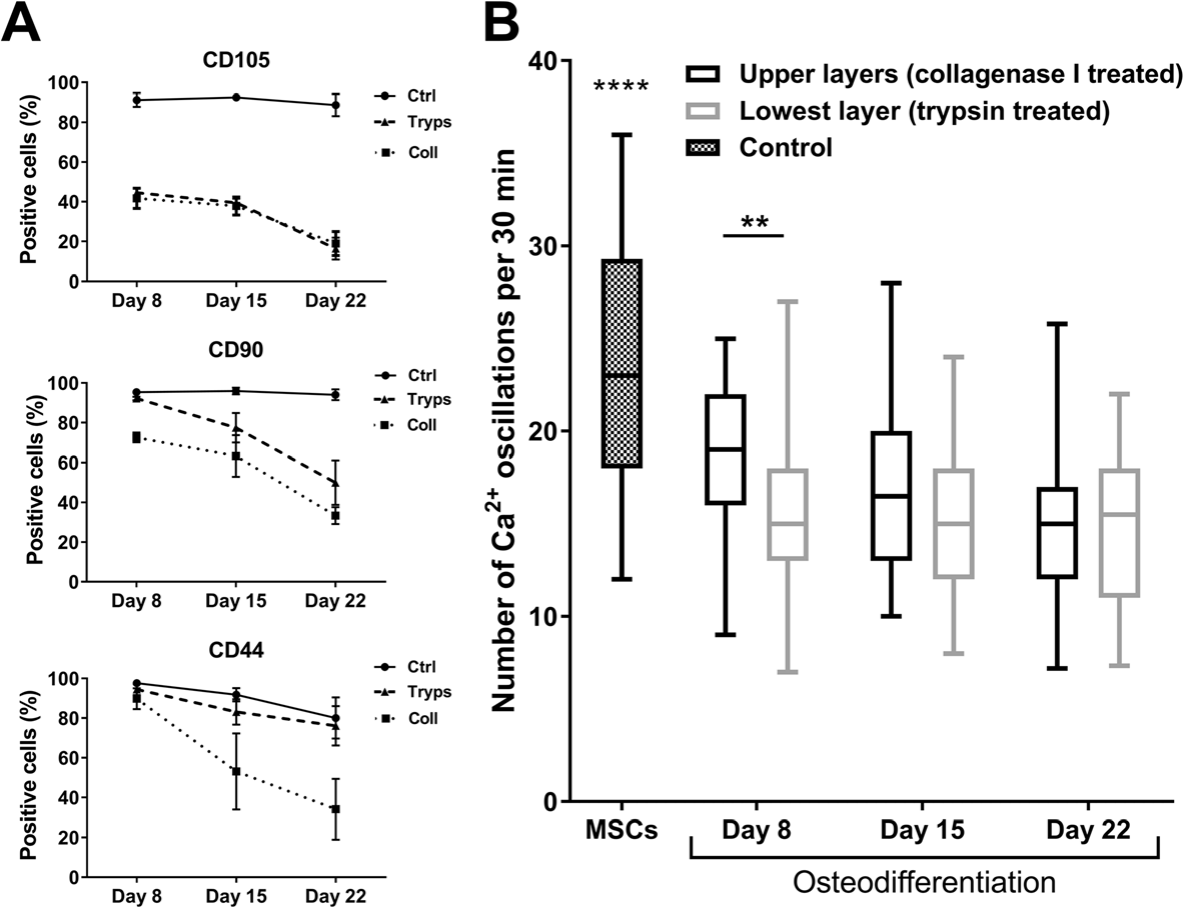
**a** Immunostaining of upper and lowest layers in osteogenic differentiation. **b** Comparison of number of Ca^2+^ oscillations between upper and lowest layers on different days of osteogenic differentiation. haMSC controls (undifferentiated haMSCs grown in classical MSC culture medium) have significantly more Ca^2+^ oscillations than differentiated layers (****p < 0.0001, one-way ANOVA with Dunnett’s multiple comparisons test). At day 8, Ca^2+^ oscillations were significantly lower in number in lowest layer than in upper layers (**p < 0.01, *p* = 0. 0038, two-way ANOVA with Sidak’s multiple comparisons test). Whiskers represent 95th and 5th percentiles. Ctrl control, tryps lowest layer trypsinized, Coll upper layers dissociated with collagenase I, MSC mesenchymal stem cell

The upper (collagenase sensitive) and lowest (trypsin sensitive) layers were labeled using MSC markers at days 1, 8, 15 and 22 (Fig. 5a). As expected, the cells significantly lost the MSC markers (CD105, CD90 and CD44) when they progressed in the differentiation (*p* < 0.0001, two-way ANOVA). Unexpectedly, this immunostaining showed different evolutions between the upper and the lowest layers. The CD105 expression was the most rapidly lost among the three. At day 8, 60% of the cells had already significantly lost it (*p* < 0.0001, two-way ANOVA with Tukey’s multiple comparisons test). At day 22, only 15–20% of the cells were still positive for CD105 labeling. No significant differences between the upper and lowest layers were observed in terms of CD105 labeling (*p* = 0.7081, *p* = 0.8878 and *p* = 0.7531 at days 8, 15 and 22, two-way ANOVA with Tukey’s multiple comparisons test). The loss of the CD90 and CD44 positive labeling was slower and more progressive (Fig. 5a). Moreover, a different evolution was found in the cells from the upper and lowest layers. The decrease was significantly faster in the trypsin-treated cells (lowest layer) than in the collagen-treated cells (upper layers) (*p* < 0.05 to *p* < 0.001, two-way ANOVA with Tukey’s multiple comparisons test), except for day 8 for CD44 (*p* = 0.8376, two-way ANOVA with Tukey’s multiple comparisons test). The cells in the controls expressed their markers at high levels all of the time.

The different layers were also compared in terms of cytosolic Ca^2+^ oscillations. Indeed, it was shown that the frequency of spontaneous Ca^2+^ oscillations in MSCs decreases rapidly with osteodifferentiation to the level observed in terminally differentiated human osteoblasts [27]. Therefore, the Ca^2+^ oscillation frequency was studied for the different cell layers in order to compare their osteogenic level of differentiation. These oscillations significantly decreased when the haMSCs were put in the osteodifferentiation medium (*p* < 0.0001, one-way ANOVA with Dunnett’s multiple comparisons test) (difference between haMSCs and cells under differentiation shown in Fig. 5b). At all of the days observed, the mean Ca^2+^ oscillations were lower in number (per 30 min) in the lowest layer than in the upper layers, even though this is only significant at day 8 (*p* < 0.01, *p* = 0.0038, two-way ANOVA with Sidak’s multiple comparisons test).

**Fig. 6:**
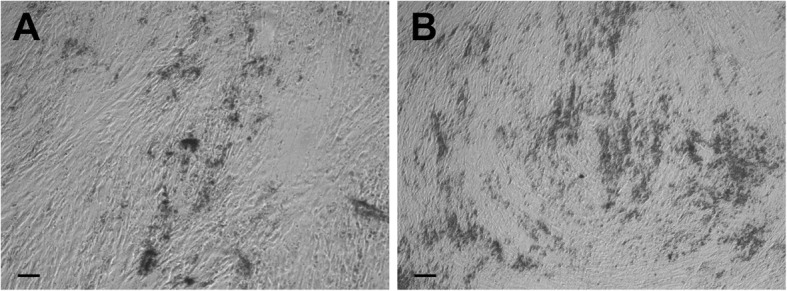
Cells from lowest (**a**) and upper (**b**) layers dissociated on day 15 and put in osteogenic medium for 2 weeks. Black spots, Ca^2+^ deposits. Calibration bars correspond to 100 μm

At day 15, cells from the upper layers (dissociated by collagenase) and the lowest layer (dissociated by trypsin) were put in culture separately in osteogenic medium. Both cell types recovered cell proliferation (Additional file 1: Figure S1), and after 2 weeks they formed two or three of cells with Ca^2+^ deposits (data not shown). The mineralization is less important in the cells differentiated from the lowest layer (Fig. 6a) than in the cells differentiated from the upper layers (Fig. 6b) (Additional file 2: Figure S2). The cells of the upper and lowest layers placed in culture with a normal medium (without osteoinductive factors) did not form Ca^2+^ deposits (data not shown).

## Discussion

### Existence of distinct layers in haMSC osteogenic differentiation

In our study, we performed for the first time a sequential treatment to dissociate haMSCs undergoing osteogenic differentiation in 2D in-vitro cultures, first using collagenase I and then trypsin. Collagenase I is used to dissociate cells from rodent or human bones in order to obtain primary osteoblasts [28, 29]. Collagenase I, at a high concentration (20 mg/ml) and with agitation at 37 °C, is also used in 3D cultures in vitro, for example to harvest cells from microcarrier cultures [25]. Collagenase B was also reported to dissolve collagen scaffolds in in-vitro 3D cultures [24]. In the work reported here, haMSCs were differentiated into osteoblasts. The multipotency of these cells was also assessed by their ability to differentiate into adipocytes and neuronal cells (as shown in Fig. 1). The use of collagenase I did not dissociate all of the cells in the osteogenic differentiation since a cell layer remained attached to the bottom of the dish as shown in Fig. 4. To the best of our knowledge, only a single study by Franceschi et al. [30] mentioned a “remaining layer”. They used 0.15 M phosphate buffer containing 2 mM phenylmethylsulfonylfluoride and 2.5 m M *N*-ethylmaleimide to dissociate the cells (until day 7 of osteodifferentiation). After that, they collected the cells of the remaining layer separately. The remaining layer in our case had to be trypsinized. Our results demonstrate that there are two types of layers in osteogenic differentiation that have not been explored previously: the upper layers which are collagenase I sensitive; and the lowest layer, attached to the substrate, which is trypsin sensitive only. It must be noted that in our experiments the use of trypsin only (until day 15) showed an effect on all of the layers and detached all of the cells, but the cells were not as well dissociated as with the collagenase I (data not shown). We will thus refer to the upper layers as the collagenase-sensitive layers and to the lowest layer as a trypsin-sensitive layer.

As described in Results, at day 22 the cells formed four layers in osteogenic differentiation, one layer in neurogenic differentiation and two layers, one continuous and one discontinuous, in adipogenic differentiation. Thus, only the cells in osteogenic differentiation form several continuous layers. These differences in the number of cell layers cannot be solely attributed to the presence of ascorbic acid in the osteogenic medium and its role in extracellular matrix synthesis. Indeed, the adipogenic differentiation (achieved in the absence of ascorbic acid) generated two cell layers. On the other hand, these observations correlate perfectly with the evolution of the cell number, since the cells proliferated massively for the first 15 days in osteogenic differentiation and this proliferation was higher than in the adipogenic and neurogenic differentiations. After day 15, we observed no further proliferation of the cells as well as the beginning of the mineralization. The evolution of the cell number in osteogenic differentiation corroborates the observations in other studies showing also that cell proliferation stops by the second week of the osteogenic differentiation [3]. Cells have to proliferate before mineralization in order to increase bone mass.

After mineralization started, the action of collagenase I was more difficult. This is why we first treated the cells with 20 mM EDTA to remove the Ca^2+^ deposits. EDTA is classically used during osteoblast isolation from rodent and human bones [28]. EDTA is also used in vitro on fixed cells in order to remove Ca^2+^ deposits [9] or combined with trypsin for harvesting cells in osteogenic differentiation [24, 25]. However, the EDTA concentrations used in vitro on fixed cells (about 350 mM) were 9–17 times higher than the concentrations we used here on viable cells [9]. The dissociation of the upper and lowest layers on day 29 required a higher EDTA concentration (40 mM) and a longer treatment time (40 min). After this intense EDTA treatment, collagenase I dissociated and detached all of the cells from the dish, probably because EDTA weakened the bonds between the lowest layer and the bottom of the dish due to the chelation of the Ca^2+^ necessary to keep the cells attached to the culture substrate.

### Differences between upper and lowest layers

The upper and lowest layers were characterized by cluster of differentiation (CD) expression and by Ca^2+^ oscillations. The MSCs express CD105, CD90 and CD44 when they are multipotent [31, 32]. As the cells progress in the differentiation process, they lose the multipotency markers compared to the control cells. The two types of layers showed no difference in losing the CD105 expression but showed a difference in the CD90 and CD44 evolution. This observation reveals that the cells in the two layers are not identical. Since the decrease was probably faster in the cells of the upper layer, these latter could be considered more differentiated than the cells of the lowest layer. The determination of the number of spontaneous Ca^2+^ oscillations during a given period of time confirmed previous results showing that the frequency of Ca^2+^ oscillations decreases progressively when MSCs undergo the osteogenic differentiation [27]. It also revealed, again, that cells of the two layers behave differently. On day 8 of osteodifferentiation, the Ca^2+^ oscillation frequency was lower in the lowest layer than in the upper layers, which could indicate that the cells of the lowest layer could differentiate faster than the cells of the upper layer. However, no significant difference was observed on days 15 and 22.

To further investigate the osteogenic potential of the two layer types, cells from the upper layers and cells from the lowest layer were taken at day 15 and separately put again in the osteogenic medium at the same confluency as at day 1 of osteodifferentiation. Cell growth resumed and after 2 weeks cells had again formed multilayers as well as Ca^2+^ deposits. The Ca^2+^ deposits were more extensive in the cells from the upper layers. Moreover, the cells originated from lowest layer proliferated more extensively than the cells originated from the upper layers. Three conclusions can be drawn from this experiment: the first is that the cells, even on day 15, could proliferate again provide they are separated and cultured again. It is true that they did not form four layers as in the first 2 weeks of osteodifferentiation but they still formed two or three layers of cells, repetitively. On the contrary, if the layers had remained together, they would have stopped proliferation at day 15 (Fig. 2). The second conclusion is that this experiment confirmed our previous observations showing that the two cell layer types are different. The upper layers are slightly more differentiated than the lowest one. Indeed, the former cells proliferated less than the cells originated from the lowest layer and they produced more Ca^2+^ deposits after a further 2 weeks. The cells located in the upper layers are embedded in the collagen matrix that they produce, which could facilitate their osteogenic differentiation [33]. Therefore, the use of collagen-coated plates might reduce the differences observed between the upper and lower layers. However, access to oxygen and to the differentiation factors present in the medium might also be limited in the lower layer, which could also contribute to their lower differentiation. Finally, the very important role of the osteogenic extracellular matrix is confirmed. As mentioned previously, osteoblasts must be in contact with such a matrix to differentiate [4, 14, 34]. When cells were dissociated and separated from the extracellular matrix, as in our case, the cells again presented an ability to proliferate.

## Conclusions

We show that haMSCs undergoing osteogenic differentiation form several layers. Collagenase I was used to dissociate the cells of the upper layers, which gives access to a lowest attached layer sensitive to trypsin. The collagenase-sensitive upper layers and the trypsin-sensitive lowest layer showed differences in the expression of the haMSC CDs. The characterization of these two types of layers that are not identical constitutes a new observation in osteogenic differentiation which offers new possibilities in the control of the number, the quality and the time to produce terminally differentiated osteoblasts. We can suggest that the investigators could indeed, on the one hand, dissociate the upper layers by collagenase I on day 15, expand them and use them rapidly (in 15 days) as terminally differentiated osteoblasts; and on the other hand, treat the lowest layer with trypsin and again put the cells in culture to keep them in proliferation, to dissociate them later and to continue the cycle of the production of differentiated osteoblast in only 2 weeks (instead of 4 weeks) from a pool of rapidly growing cells in the middle of the differentiation process. This cycle should favor both the production of differentiated osteoblasts and the maintenance of a stock of cells undergoing the differentiation facilitating the application of these cells and spreading their uses.

## Additional files

Additional file 1: **Figure S1.** Cell growth in osteodifferentiation medium for three consecutive periods of 15 days. Culture dishes always seeded with same number of cells at day 1 and cells counted after 15 days of culture. Growth reported as increase fold in number of cells at day 15. haMSCs cultured in osteodifferentiation medium for “first differentiation”. After 15 days, upper layers (U1) and lowest layer (L1) were counted and seeded separately in osteodifferentiation medium (“second differentiation”). After 15 more days, upper layers (U2 from U1 and U2 from L1) and lowest layer (L2 from U1 and L2 from L1) were counted and seeded separately in osteodifferentiation medium (“third differentiation”). After 15 days, all cells were counted for each condition (U2(U1), U2(L1), L2(U1) and L2(L1)). Each condition counted three times in three independent repeats. Diff differentiation. (TIF 117 kb)

Additional file 2: **Figure S2.** Quantification of Alizarin Red staining in upper and lowest layers. haMSCs were cultured in osteodifferentiation medium for “first differentiation”. After 15 days, upper layers (U1) and lowest layer (L1) were seeded separately in osteodifferentiation medium (“second differentiation”). After 15 more days, upper layers (U2 from U1 and U2 from L1) and lowest layer (L2 from U1 and L2 from L1) were seeded separately in osteodifferentiation medium (“third differentiation”). After 15 days, for each condition (U2(U1), U2(L1), L2(U1) and L2(L1)), deposits of calcium phosphate were stained with Alizarin Red and quantified by elution of stain using cetylperidinium chloride and quantification by spectrophotometry. Results normalized by number of cells. Each condition quantified three times in three independent repeats. (TIF 125 kb)

## Abbreviations

ALP: Alkaline phosphatase
CD: Cluster of differentiation
DMEM: Dulbecco’s Modified Eagle Medium
ECM: Extracellular matrix
FABP4: Fatty acid binding protein 4
haMSC: Human adipose mesenchymal stem cell
MAP2: Microtubule-associated protein 2
MSC: Mesenchymal stem cell
pNP: *p*-Nitrophenol
pNPP: *p*-Nitrophenyl phosphate
